# Asymptomatic Herpes Simplex Virus Type 1 Infection Causes an Earlier Onset and More Severe Experimental Autoimmune Encephalomyelitis

**DOI:** 10.3389/fimmu.2021.635257

**Published:** 2021-02-15

**Authors:** Luisa F. Duarte, María J. Altamirano-Lagos, Jorge H. Tabares-Guevara, Ma. Cecilia Opazo, Máximo Díaz, Romina Navarrete, Catalina Muza, Omar P. Vallejos, Claudia A. Riedel, Susan M. Bueno, Alexis M. Kalergis, Pablo A. González

**Affiliations:** ^1^Millennium Institute on Immunology and Immunotherapy, Santiago, Chile; ^2^Departamento de Genética Molecular y Microbiología, Facultad de Ciencias Biológicas, Pontificia Universidad Católica de Chile, Santiago, Chile; ^3^Departamento de Ciencias Biológicas, Facultad de Ciencias de la Vida, Universidad Andrés Bello, Santiago, Chile; ^4^Departamento de Endocrinología, Facultad de Medicina, Escuela de Medicina, Pontificia Universidad Católica de Chile, Santiago, Chile

**Keywords:** HSV-1, viral infection, multiple sclerosis, experimental autoimmune encephalomyelitis, neuroinflammation

## Abstract

Multiple sclerosis (MS) is an increasingly prevalent progressive autoimmune and debilitating chronic disease that involves the detrimental recognition of central nervous system (CNS) antigens by the immune system. Although significant progress has been made in the last decades on the biology of MS and the identification of novel therapies to treat its symptoms, the etiology of this disease remains unknown. However, recent studies have suggested that viral infections may contribute to disease onset. Interestingly, a potential association between herpes simplex virus type 1 (HSV-1) infection and MS has been reported, yet a direct relationship among both has not been conclusively demonstrated. Experimental autoimmune encephalomyelitis (EAE) recapitulates several aspects of MS in humans and is widely used to study this disease. Here, we evaluated the effect of asymptomatic brain infection by HSV-1 on the onset and severity of EAE in C57BL/6 mice. We also evaluated the effect of infection with an HSV-1-mutant that is attenuated in neurovirulence and does not cause encephalitis. Importantly, we observed more severe EAE in mice previously infected either, with the wild-type (WT) or the mutant HSV-1, as compared to uninfected control mice. Also, earlier EAE onset was seen after WT virus inoculation. These findings support the notion that a previous exposure to HSV-1 can accelerate and enhance EAE, which suggests a potential contribution of asymptomatic HSV-1 to the onset and severity of MS.

## Introduction

Multiple sclerosis (MS) is an autoimmune inflammatory disorder of the central nervous system (CNS) that affects both, the brain and spinal cord in which multifocal autoreactive lymphocytic infiltrations lead to the damage of the myelin and the axons of neurons (1, 2). Defining what triggers the loss of immunologic tolerance to CNS antigens and contributes to the onset of autoreactivity with infiltration into these tissues remains elusive (3, 4). Likely, MS develops as an interplay between genetic predisposition, the immune system and environmental factors, among which viral infections may contribute to its onset and severity (5).

Herpes simplex virus type 1 (HSV-1) infection is highly prevalent in the human population with nearly two thirds of the world population infected with this virus (6). HSV-1 is neurotropic and causes a wide spectrum of clinical manifestations, ranging from mild symptoms such as oral and facial lesions (e.g., herpetic gingivostomatitis and *herpes labialis*), to more severe diseases affecting the eyes and CNS (e.g. herpetic keratitis, retinitis, encephalitis, and meningitis) (7, 8). Importantly, HSV-1 can access the CNS with no apparent pathology (asymptomatic) establishing a persistent latent infection in these tissues (9). Accumulating evidence indicates that healthy individuals frequently have HSV-1 DNA in the brain, which could eventually relate to, and favor the development or enhancement of the severity of neurodegenerative disorders by altering normal neuronal cell functions due to subclinical HSV-1 reactivations within CNS neurons (10, 11). HSV-1 may also contribute to local and regional dissemination of the virus, as well as long-term detrimental effects to this tissue (12, 13). Importantly, HSV-1 infection of the CNS is characterized by persistent lymphocytic cell infiltrations and elevated levels of cytokine transcripts (e.g., IFN-γ, TNF-α), as well as increased amounts of chemokine mRNAs (e.g., CXCL10, CCL5), suggesting that latent HSV-1 infection can be accompanied by a chronic inflammatory process in this tissue (13–15). Moreover, increased levels of the matrix metalloproteinases 2 and 9 (MMP-2 and MMP-9) have been detected in HSV-1 latently-infected CNS, which could contribute to the degradation of the surrounding extracellular matrix and cell surface proteins leading to a partial breakdown of the blood-brain barrier (BBB), which plays an important role in MS (16, 17). This inflammatory response could arise due to low-level expression of viral genes during HSV-1 latency of the CNS (18), which could promote or facilitate an inflammatory environment that modulates the onset and severity of neurological disorders (12, 19).

Importantly, viruses belonging to the *Herpesviridae* family have been suggested as potential triggers and positive modulators of MS (20). For instance, the human herpesvirus 6 (HHV-6) was recently shown to increase the severity of MS-like symptoms in non-human primates treated to undergo experimental autoimmune encephalomyelitis (EAE) (21). In another study, latent-infection with the homologous of Epstein-Barr virus in mice (γHV-68 virus), prior to EAE induction was shown to enhance the pathogenesis of active EAE, which was associated with increased CD4^+^ and CD8^+^ T cell responses in the brain and spinal cord, yet was independent of viral reactivation (22, 23). On the other hand, a study performed in rats showed that repeated inoculations with HSV-1 elicited clinical and histological evidence of exacerbated EAE, but the possible mechanisms behind this observation were not determined (24). Additionally, HSV-1 genetic material has been found more frequently in the cerebrospinal fluid and blood of MS patients than control subjects, suggesting an association between this virus and MS (25–27). However, a direct relationship between both, as well as the potential mechanisms underlying a role for HSV-1 over MS, or vice-versa have not been elucidated. Here, we assessed whether sub-lethal infection of the CNS with HSV-1 that produces asymptomatic infection in the mouse modulates the severity of MS-like symptoms upon the induction of EAE, which is widely used as a surrogate model for multiple sclerosis. Importantly, we used C57BL/6 mice, which are resistant to HSV-1 acute brain infection and to HSV-1-induced demyelinating lesions throughout the brain (28), to facilitate the assessment of asymptomatic brain infection by HSV-1 over EAE disease. We also performed experiments with an HSV-1 mutant virus that has the gamma-34.5 gene (*ICP34.5*) deleted (29). This mutant virus has been reported to replicate in peripheral tissues, but is attenuated in neurons and does not cause encephalitis (29).

Interestingly, we found that infection with either virus elicited prolonged alterations to the BBB, which may account for some of the effects described below. We found that HSV-1 infection with the WT virus accelerated the onset of EAE. Furthermore, a previous infection with either, the WT virus or the attenuated mutant virus elicited a more severe EAE disease in mice, which was accompanied by increased CNS inflammation, as well as histological alterations in the related tissues. Additionally, infected animals induced to undergo EAE showed an increase in activated microglia in the brain and spinal cord, more infiltrating CD4^+^T cells in the brain and higher amounts of neutrophils in the spinal cord. We also found significantly higher levels of IL-6, TNF-α, and IL-1β mRNA in these tissues. Taken together, our results suggest a direct relationship between asymptomatic HSV-1 infection after intranasal viral inoculation and an increased susceptibility to undergo a more severe form of EAE. The implications of these findings are discussed.

## Materials and Methods

### Mice and Viruses

Five-week-old C57BL/6 female mice were obtained from The Jackson Laboratory (Bar Harbor) and maintained with environment enrichment, sterile food and water *ad libitum* at the central animal facility of the Pontificia Universidad Católica de Chile. Virus stocks were prepared and titers were determined in Vero cells (ATCC® CCL-81) and kept at −80°C until use. WT HSV-1 (17syn+ strain) and the R3616 HSV-1 mutant used in this study were kindly provided by Dr. Carola Otth (Universidad Austral de Chile, Chile). R3616 lacks the gamma-34.5 gene *(*Δ*ICP34.5*) and was generated from HSV-1 strain F and generously donated, through Dr. Otth, by Dr. Bernard Roizman (University of Chicago, USA) (30). All procedures in this study were approved by the Scientific Ethical Committee for Animal and Environmental Care of the Pontificia Universidad Católica de Chile and the Biosafety Committee of the same institution (Protocol #170705018) and were performed according to the National Institutes of Health Guide for Care and Use of Animals (31).

### Infections and EAE Induction

Five-weeks-old C57BL/6 female mice were infected intranasally with a sub-lethal dose of 10^6^ plaque forming units (PFU) of WT (17syn+) or Δ34.5 (F) HSV-1, as previously described (32, 33). Mock-treated mice were used as controls, which were inoculated with supernatant obtained from Vero cells cultures. During the first 2 weeks post-infection, mice were clinically scored daily for neurological symptoms as follows: Normal (0), ataxia (1), hunched posture (1), forelimbs paralyzed yet mobile-capable (1), forelimbs paralyzed and immobility (2), seizures or circling (1). The scores corresponding to each symptom are added and the final clinical score is accumulated. The experimental approach undertaken elicits asymptomatic HSV-1 infection without clinical manifestations of encephalitis. EAE was induced 30–35 days post-infection after asymptomatic HSV-1 infection. Briefly, mice were anesthetized with a mixture of ketamine (80 mg/kg) and xylazine (4 mg/kg) injected subcutaneously with 50 μg of myelin oligodendrocyte glycoprotein-(MOG)-derived peptide (MOG_35−55_, sequence MEVGWYRSPFSRVVHLYRNGK; Pan Web, Stanford University) emulsified in complete Freund's adjuvant (Thermo Fisher Scientific) supplemented with heat-inactivated *Mycobacterium tuberculosis* H37 RA (DIFCO). Mice also received two intraperitoneal injections of 350 ng of pertussis toxin (List Biological Laboratories, Inc.) at the time of induction and 48 h later. Mice were scored daily based on an EAE scale as follows: 0, no changes in motor function; 0.5, the tip of the tail is limp; 1, limp tail; 2, limp tail and weakness of hind legs; 2.5, limp tail, and one hind limb paralyzed; 3, limp tail, and complete paralysis of hind limbs; 3.5, hind limbs and one fore limb paralyzed; 4, hind limbs and forelimbs completely paralyzed; 5, moribund.

### Blood-Brain Barrier Integrity Assay

The integrity of the blood brain barrier (BBB) of HSV-1-infected mice was evaluated using an Evans blue (EB, Sigma-Aldrich) dye exclusion test, as previously reported (34). Thirty days post-infection, mice were transcardially perfused with 50 mL of phosphate-buffered saline (PBS, pH 7.4), followed by 50 mL of the EB 2% in PBS under lethal ketamine/xylazine dose. Brains and spinal cords were dissected, fixed in 4% of p-formaldehyde (PFA), and cryopreserved in PBS with 30% sucrose for 24 h. Later, organs were embedded in cryostat-embedding compound (OCT, Sakura), cut into 20 μm thick sections on a cryostat at −22°C and mounted on Superfrost slides (Thomas Fisher Scientific). Slides were examined under a confocal laser microscope (Leica TCS LSI), and EB extravasation was visualized as red fluorescence using a 543-nm laser. Additionally, the amount of EB entering the CNS was quantified by spectrophotometry at 620 nm after tissue homogenization in 50% of trichloroacetic acid in PBS and normalized according to the weight of the tissue (EB ng/mg tissue) (35).

### Histological Analyses and Immunohistochemistry

Mice infected with HSV-1 and induced to develop EAE were transcardially perfused with 50 mL of PBS to remove intravascular leukocytes. Lumbar regions in the spinal cords and corpus callosum in the brain were dissected and carefully processed for histological analyses at day 14, 21, and 25 post-EAE induction. Briefly, tissues were fixed for 24 h in 4% PFA, dehydrated with ethanol and embedded in paraffin. Six micrometer thick sections were obtained using a microtome, and slices were stained with Luxol Fast Blue solution (LFB) (0.1%, 2 h at 60°C) and counterstained with Cresyl violet (0.1%, 6 min) to evaluate demyelination and cell infiltrates, respectively. Four to five sections per mice were analyzed using an Axio Vert.A1 microscope (Zeiss) with a 10X and a 20X objective and histopathologic scores were determined as follows: 0, no inflammation or demyelination; 1, one inflammation focus with slight demyelination and/or with few infiltrates; 2, two inflammation foci with moderate demyelination and/or moderate increase in infiltrates; 3, three or more inflammation foci with severe or complete demyelination and/or extensive cell infiltration, as previously described (36). Additionally, immunohistochemistry against the myelin basic protein (MBP) was carried out using the Mouse-on-Mouse HRP-Polymer Bundle kit (Biocare Medical). The procedure was carried out following the manufacturer's instructions. Briefly, sections were deparaffinized with xylene and rehydrated with decreasing concentrations of alcohol. Endogenous peroxidase was quenched with 3% H_2_O_2_ in PBS for 20 min, followed by several washes in PBS. Antigen retrieval was performed using the reagent Rodent Decloaker 1X (Biocare medical) at 95°C for 40 min in a steamer. Then, slides were incubated for 30 min at room temperature (RT) in Rodent Block M (Biocare medical) for 30 min, followed by 60 min incubation at 37°C with a dilution 1:1,000 of primary anti-MBP antibody (SMI-99P, Biolegend) in 1% bovine serum albumin (BSA, Winkler) in PBS and 0.1% Triton X-100. After washes with PBS pH 7.4, Mouse-on-Mouse HRP-Polymer was added for 30 min. Finally, immunostaining was performed using 0.05% diaminobenzidine and 0.015% H_2_O_2_ and counterstained with hematoxylin for 5 min. Slides without primary antibody were used as controls.

### Western Blot Analyses

Western blot analyses were performed to evaluate the expression of MBP in lumbar regions in the spinal cord and corpus callosum in the brain of mice latently infected with HSV-1 and induced to develop EAE at day 14 and 21 post-EAE induction. Samples were homogenized, placed in lysis buffer (150 mM NaCl, 1 mM EDTA, 10 mM Tris-HCl, 1 mM phenylmethanesulfonyl fluoride, 0.5% NP40, 0.5% Sodium Deoxicholate, and 0.1% SDS), and total protein was determined using the Pierce BCA Protein Assay Kit (Thermo Fisher Scientific) following the manufacturer's instructions. Proteins were resolved using 12% sodium dodecyl sulfate (SDS) polyacrylamide gel electrophoresis and transferred to nitrocellulose membranes (Bio-Rad). After blocking with 5% BSA, membranes were incubated overnight at 4°C with a 1:300 dilution of mouse anti-MBP (SMI-99P, Biolegend) or a 1:1,000 dilution of anti-β-actin (2F1-1, Biolegend) for 2 h at RT. A horseradish peroxidase (HRP)-conjugated anti-mouse antibody was used as secondary antibody (GenScript), and proteins were visualized by chemiluminescence using a ChemiDoc®MP Imaging System (Bio-Rad). Band intensity was calculated using ImageJ (U.S. National Institutes of Health).

### Mononuclear Cell Isolation, Staining, and Flow Cytometry

Single cell suspensions were generated at day 14 post-EAE induction from the spinal cord and brain of HSV-1-infected EAE-induced mice perfused with PBS, as previously reported (37). Infected and uninfected mice without EAE were used as controls, which were euthanized at equivalent time-points than mice with EAE (6 weeks post-infection). Tissues were incubated with 1 mg/mL collagenase IV (Thermo Fisher Scientific) and 50 μg/mL DNAse I (Roche) in RPMI (Thermo Fisher Scientific) at 37°C for 30 min. Mononuclear cells (MNCs) were isolated using 30/70% Percoll gradients (GE healthcare). For staining, MNCs were treated with CD16/32 Fc-block (BD Biosciences) to inhibit nonspecific antibody binding and incubated with anti-mouse immune cell surface markers for 45 min at 4°C. The following antibodies were used: anti-CD3 (Clone 17A2), anti-CD4 (clone 6K1.5), anti-CD8 (clone 53-6.7), anti-CD19 (clone 1D3), anti-CD45 (clone 30-F11), anti-CD11b (clone M1/70), anti-Ly6C (clone HK 1.4), anti-Ly6G (clone RB6-8C5), and anti-MHC-II (clone AF6-120.1) (BioLegend). Dead cells were detected using the fixable Zombie Violet kit (BioLegend) and excluded from the analyses. Cells were enumerated by adding CountBright™ absolute counting beads (Thermo Fisher Scientific), to each sample before acquisition using a FACSCanto II flow cytometer (BD Biosciences) and data was analyzed using FlowJo software (Tree Star, Inc.). This work was supported by the Flow Cytometry Core UC (FCC UC).

### Quantitative PCR (qPCR) and Reverse Transcription Quantitative PCR (RT-qPCR)

Total DNA from the brain and trigeminal ganglia tissues was isolated at day 30 or 45 post-HSV-1 infection, or at day 15 post EAE induction by using phenol-chloroform (Winkler) for quantifying the number of viral genomes. Two hundred nanograms of DNA was used for qPCR analysis with the following primers and probe for the viral polymerase *UL30* gene: Fwd-GGCCAGGCGCTTGTTGGTGTA, Rev-ATCACCGACCCGGAGAGGGA and Probe-CCGCCGAACTGAGCAGACACCCGC (Integrated DNA Technologies) and an Applied Biosystems StepOnePlus thermocycler, as previously described (38). Total RNA was isolated from the tissues for cytokine expression analyses at day 14 post-EAE induction by using TRIzol reagent (Thermo Fisher Scientific) according to the manufacturer's instructions. RT-qPCR reactions were carried out using TaqMan® RNA-to-Ct™ 1-Step Kit (Thermo Fisher Scientific) and TaqMan® probes for the detection of IL-1β (Ref: Mm00434228_m1), IFN-γ (Ref: Mm 01168134_m1), TNF-α (Ref: Mm04204156_gH), IL-10 (Ref: Mm00439614_m1), IL-6 (Ref: PN 4331348), and β-actin (Ref: Mm02619580_g1) on the StepOnePlus^TM^ Real-Time PCR System (Applied Biosystems®) with the following cycling conditions: one cycle at 50°C for 15 min and 95°C for 10 min, followed by 40 cycles at 95°C for 15 s, and 60°C for 1 min. The abundance of each target mRNA was determined by relative expression to the β-actin housekeeping gene and the 2^−ΔΔCT^ cycle threshold (2^−ΔΔCT^) method (39).

### ELISA Assays

Immunoglobulin G (IgG) antibodies against HSV-1 were detected by ELISA using sera obtained before and 14 days after EAE induction. MaxiSorp ELISA plates (Nunc/Thermo Scientific) were coated with 20 μg/mL of protein extracts from uninfected-Vero cells or 10 μg/mL of protein extracts from infected-Vero cells and incubated at 4°C overnight in a humidity chamber. Plates were blocked with PBS-BSA 1% and then incubated with serial dilutions of the sera. To reduce non-specific antibody binding to the infected protein extracts, the sera were pre-adsorbed over plates with uninfected-Vero protein extracts for 2 h at RT and then transferred to plates with infected-Vero protein extracts and incubated at 4°C overnight in a humidity chamber. After three washes with PBS-Tween 20 0.05%, the wells were incubated with an HRP-conjugated anti-mouse-IgG antibody diluted 1:2,000 (Thermo Fisher Scientific) for 1 h at RT, washed 3 times with PBS-Tween 20 0.05%, developed with 1-Step™ Ultra TMB-ELISA Substrate Solution (Thermo Fisher Scientific) for 10 min, and read on a Multiskan ELISA plate reader at 450 nm after adding H_2_SO_4_ 2N to stop the enzymatic reaction. Anti-MOG antibodies were also detected at day 14 post-EAE induction in the sera from uninfected-EAE and infected-EAE mice carrying out the steps mentioned above using 10 μg/mL of MOG peptide to coat the ELISA plates.

### Statistical Analyses

Statistical significance between experimental groups was assessed by *T*-test (two groups) or one-way analysis of variance (ANOVA) with Dunnett's multiple comparisons post-test for parametric data, Kruskal–Wallis with Dunn's multiple comparisons post-test for non-parametric data (three or more groups), or two-way ANOVA with Tukey's multiple comparison post-test (two independent variables) using GraphPad Prism software (GraphPad Software, La Jolla California USA).

## Results

### Asymptomatic HSV-1 Infection Alters the Permeability of the Blood Brain Barrier

To assess a potential effect of asymptomatic HSV-1 infection of the CNS over the onset and severity of EAE in the mouse model, we performed experiments with C57BL/6 mice. This mouse strain has been reported to be resistant to acute HSV-1 encephalitis and hence could better reflect circumstances related to asymptomatic and latent CNS infections reported in humans that do not show clinical manifestations, despite having the virus in the brain (11, 40). Thus, C57BL/6 mice were inoculated intranasally with a sub-lethal dose of WT HSV-1 and followed for 30 days. As expected, there were no symptoms associated to encephalitis (clinical score 0), although the weight of the animals decreased during the first 3 days after HSV-1 infection, but then recovered and increased significantly at day 30 post-infection (Supplementary Figure 1A). Additional to the use of a WT HSV-1 virus, we also included in the experiments an HSV-1 mutant that has the gene encoding the virulence factor gamma-34.5 deleted (*ICP34.5* gene) named HSV-1 Δ34.5. This mutant virus does not cause encephalitis and has been reported to be hampered at replicating in neurons, although it can elicit a chronic inflammatory response in the brain of mice, which could also somewhat homologate the case of humans undergoing asymptomatic HSV-1 infection of this tissue (33, 41). This mutant virus is impaired at establishing latency and reactivating from the nervous system (29). HSV-1 Δ34.5-inoculated animals showed a reduction in their weight 1-day post-infection, but overall paralleled the dynamics of weight variation seen for the uninfected animals (Mock-inoculated) (Supplementary Figure 1A). Latent brain infection by the WT virus was corroborated using a virus plaque assay and by qPCR 30 days post-infection. As expected, no viral PFUs were recovered from brain tissue homogenates overlaid onto Vero cells at this time-point (data not shown), while the qPCR evidenced the presence of viral genome copies both, in the trigeminal ganglia (TG) and brain of WT-infected mice, with a higher load of virus in the TG as compared to the brain (Supplementary Figure 1B). Regarding the animals inoculated with the mutant virus, viral loads at day 30 after inoculation were detected in these tissues at a low frequency and lesser amounts as compared animals that received the WT virus (Supplementary Figure 1B).

**Figure 1:**
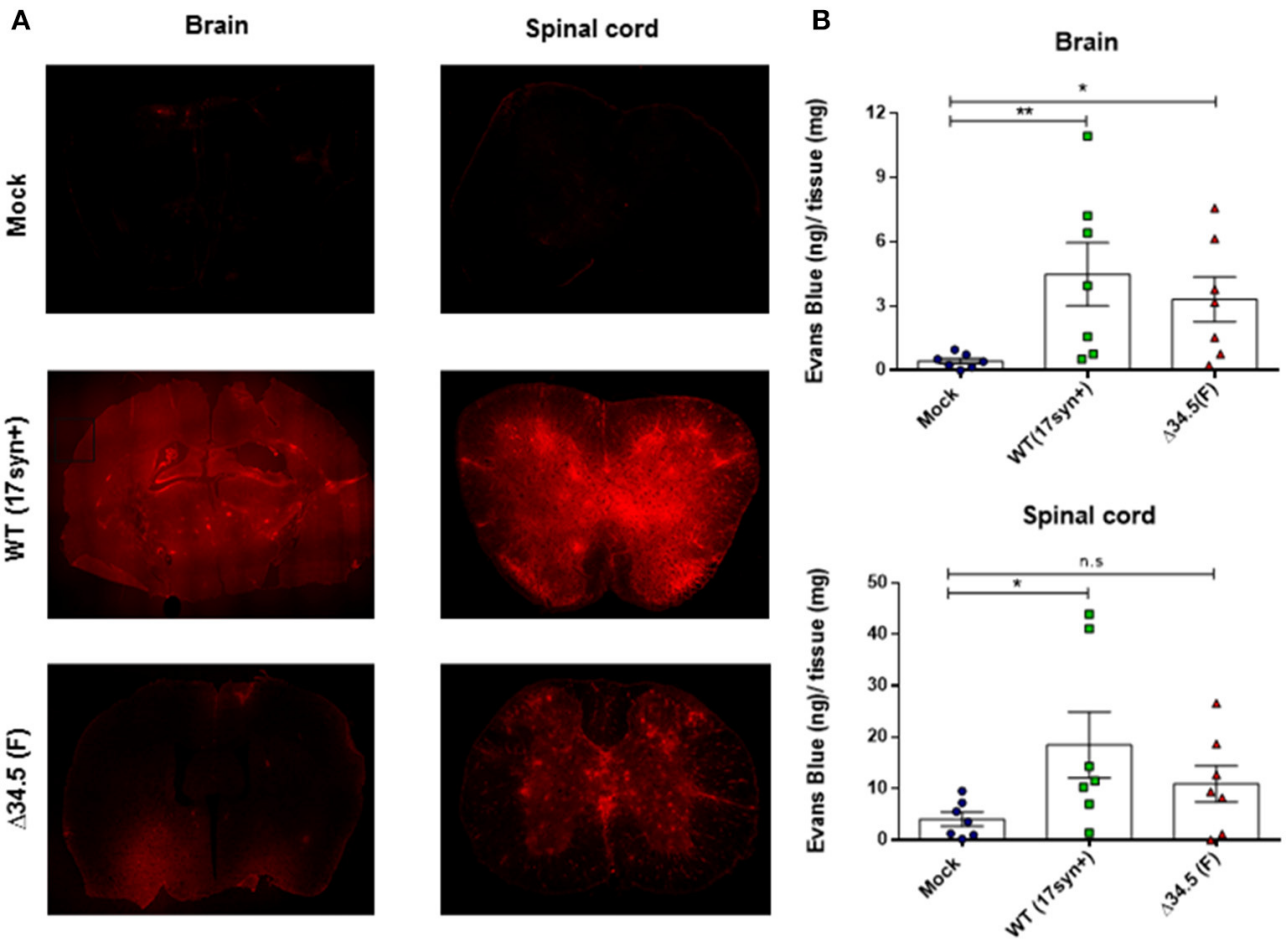
Asymptomatic HSV-1 infection increases BBB permeability *in vivo*. Thirty days post- infection mice were transcardially perfused with Evans Blue dye (2 % w/v). **(A)** Evans blue visualization by confocal microscopy in brain (left panels) and spinal cord sections (right panels) in uninfected mice or animals inoculated with WT HSV-1 (17syn+ strain) or mutant Δ34.5 HSV-1 (F strain). Representative images of two independent experiments are shown. The original magnification of the photomicrographs is 10×. The brain image is a composite of 10 serial images. **(B)** Quantification of Evans Blue dye incorporated into the brain (upper panel) and spinal cord (lower panel) by spectrophotometry at 620 nm. Values represent means ± SEM of two independent experiments (*n* = 7/group). Data were analyzed using Kruskal–Wallis and Dunn's multiple comparisons post-test; **p < 0.01; *p < 0.05.

Because previous reports indicate that acute HSV-1 infection of the brain alters the BBB (42–44), we sought to assess whether this was also the case in asymptomatic animals infected with HSV-1 30 days post-virus inoculation. For this, we used Evans blue (EB), a dye that when administered systematically cannot access the CNS in normal conditions unless the BBB is altered (34). Hence, extravasation of this dye into the CNS is indicative of increased BBB permeability. As shown in Figure 1, mice infected with WT virus presented increased EB diffusion into the brain and spinal cord at day 30 post-infection, as compared to mock-inoculated animals, suggesting that the BBB is altered in these mice long after infection and in the absence of detectable infectious virus. Notably, mice infected with the mutant HSV-1 virus also showed significantly increased EB diffusion into the brain as compared to uninfected animals, suggesting that BBB disruption is independent of viral replication in the brain. Future studies should help determine for how long the BBB is disrupted after HSV-1 infection.

### Asymptomatic HSV-1 Infection Accelerates the Onset and Increases the Severity of EAE

**Figure 2:**
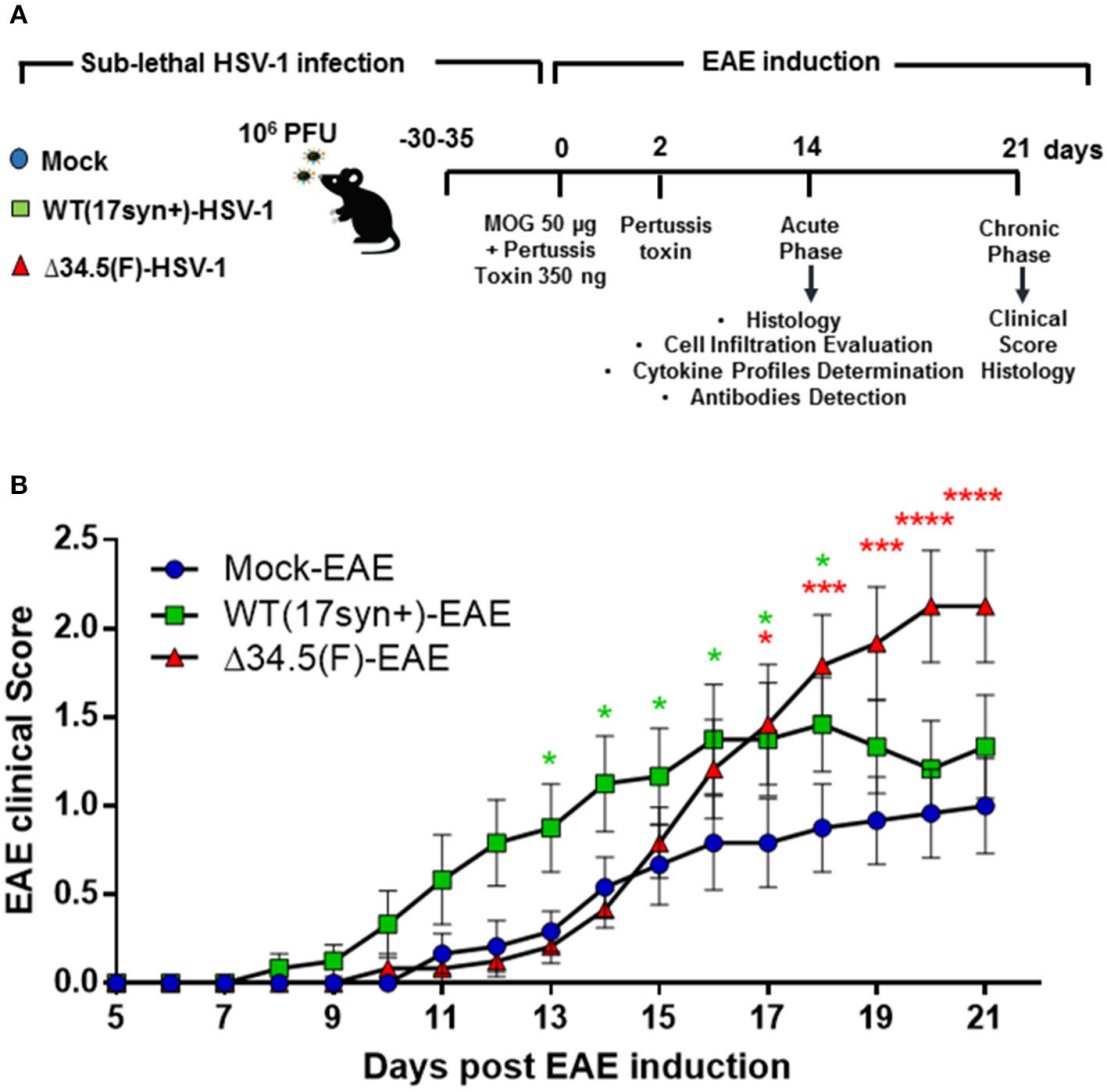
Asymptomatic HSV-1 infection accelerates the onset and increases the severity of EAE. **(A)** Schematic representation of the experimental design carried out in this study. **(B)** EAE was scored for each mouse after EAE induction, which was carried out 30–35 days post-HSV-1 infection. Mice were followed until day 21 post-EAE induction. The graph shows the means of disease scores ± SEM for mice mock-treated (blue circles), infected with WT HSV-1 (17syn+ strain, green squares) or inoculated with HSV-1 Δ34.5 (F strain, red triangles) in three independent experiments (*n* = 12/group). Data were analyzed using two-way ANOVA followed by Tukey's post-test; ****p < 0.0001, ***p < 0.001, and *p < 0.05.

To determine if HSV-1 infection impacts the onset and severity of CNS autoimmunity, we carried out an EAE induction protocol in mice that had been previously infected with HSV-1 (Figure 2A). As a control, EAE was also induced in mock-infected animals. As shown in Figure 2B, the previous infection with WT HSV-1 accelerated the onset of EAE in 2 days approximately, while infection with the Δ34.5 mutant virus displayed a similar disease onset as the mock-infected animals (Table 1). Importantly, mice infected with WT HSV-1 displayed a higher incidence and scores of EAE symptoms than non-infected animals with EAE, which started at day 12 post-induction of this autoimmune disease (Table 1 and Figure 2B). On the other hand, mice infected with the Δ34.5 mutant virus had a higher incidence and increased EAE clinical scores than WT HSV-1-inoculated animals (Table 1 and Figure 2B). Unlike the mock-EAE treated animals, which showed mild EAE symptoms, the animals infected either, with the WT or the mutant virus showed a chronic progressive course of EAE symptoms up to permanent paralysis, which would normally be observed in C57BL/6 mice after severe MOG_35−55_-induced EAE (45).

**Table 1:** Summary of EAE disease parameters after HSV-1 inoculation and EAE induction.

**Group**	**Incidence of EAE symptoms**	**Mean day of disease onset**	**Maximum clinical score of EAE**	**Mean clinical score at day 14 (disease peak)**	**Mean clinical score at day 21 (remission stage)**
Mock-EAE	66.7% (8/12)	13.6	2.5	0.5	1
WT-EAE	91.7% (11/12)	11.9	3	1.1	1.3
Δ34.5-EAE	100% (12/12)	14.1	3.5	0.4	2.1

**Figure 3:**
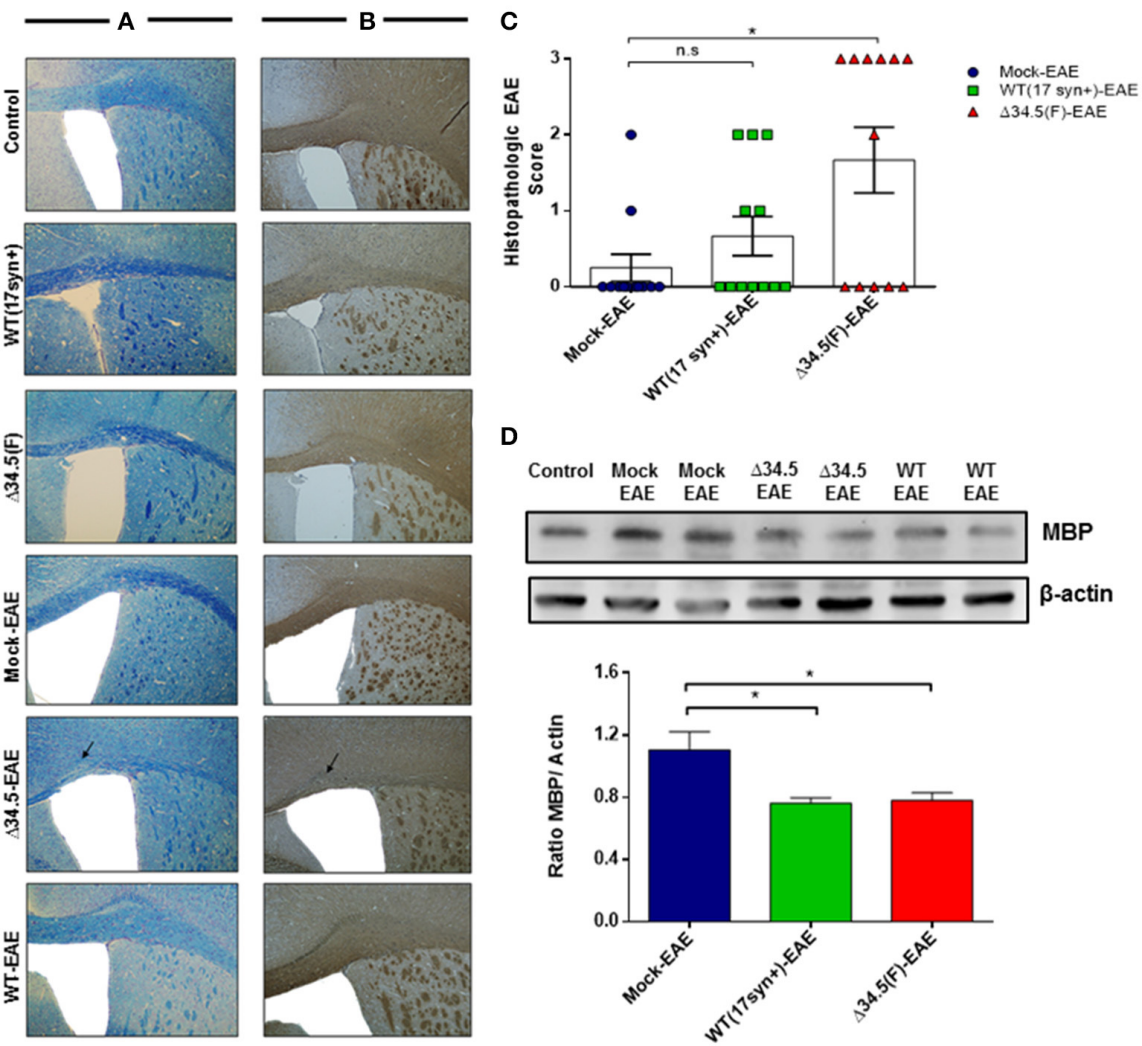
Asymptomatic HSV-1 infection contributes to brain demyelination after EAE induction. Mice were treated with mock, infected with WT HSV-1 (17syn+ strain) or inoculated with HSV-1 Δ34.5 (F strain) (*n* = 12/group). EAE was induced 30–35 days post-treatment and 4 mice from each group were euthanized at day 14, 21 or 25 post-EAE induction. **(A)** Representative images of brain sections stained with Luxol Fast Blue showing the corpus callosum. **(B)** Representative images of immunohistochemistry against the MBP protein in brain samples. The image magnification is 10× and corresponds to day 14 post-EAE induction. Arrows show demyelination sectors with reduced myelin in the corpus callosum. **(C)** Quantitative histopathological analyses of brain tissue samples. Values represent means ± SEM of three independent experiments (4 mice/group per day evaluated). Data were analyzed using one-way ANOVA with Dunnett's multiple comparison post-test; **p < 0.05 and n.s. non-significant. **(D)** Representative western blot images for MBP (upper panel) and β-actin (lower panel) in brain tissue at day 14 post-EAE induction. The graph shows densitometric analyses for MBP bands that were normalized to β-actin. Data represent the mean ± SEM of two independent experiments (*n* = 6). Comparisons between ratios were performed using one-way ANOVA with Dunnett's multiple comparison post-test; *p < 0.05.

To characterize the impact of asymptomatic HSV-1 infection on the integrity of CNS tissues after EAE induction, we performed histological and molecular analyses of brain and spinal cord samples. Histological analyses with Luxol Fast Blue, which stains the myelin was contrasted with Cresyl violet to evidence cellular infiltration. Additionally, we performed myelin basic protein (MBP) expression analyses by immunohistochemistry and western blot for this protein. As shown in the Figures 3A,B, brain tissues showed some regions of evident demyelination only in HSV-1-inoculated animals induced to develop EAE. This was not the case for HSV-1-infected only mice without EAE induction, which were used as controls. Similarly, mock-inoculated animals treated to undergo EAE did not show significant histological alterations, which was expected as the protocol used for inducing EAE in our experimental setting was mild, consistent with mild disease score values that is not associated with significant demyelination in the brain in the absence of previous viral infection (45). Moreover, HSV-1 Δ34.5-infected mice displayed significantly higher histological scores than uninfected ones (Figure 3C), and both groups infected with either, WT or the mutant virus showed lesser expression of the MBP protein by western blot as compared to mock-inoculated animals (Figure 3D).

**Figure 4:**
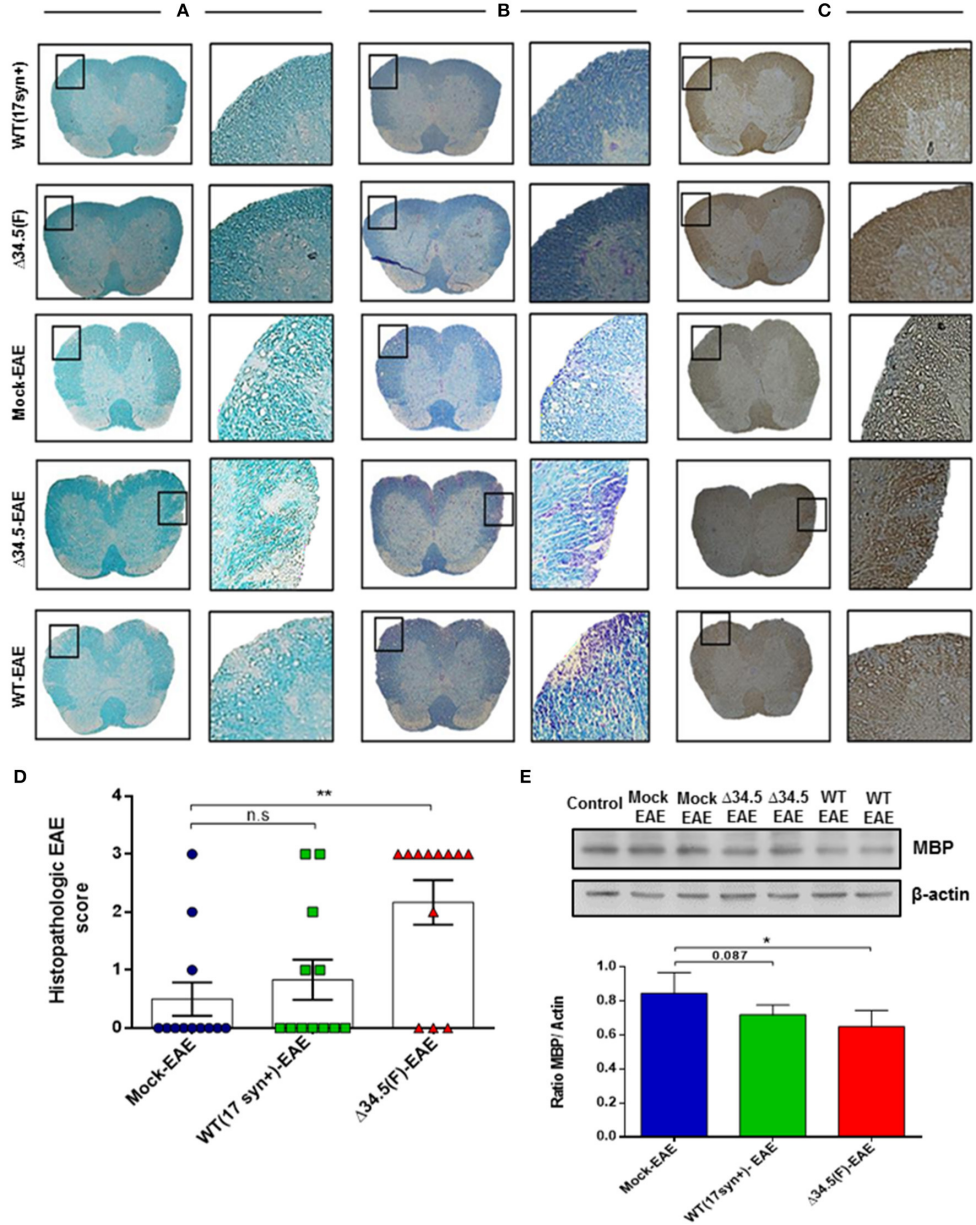
Asymptomatic HSV-1 infection increases spinal cord demyelination after EAE induction. Mice were treated with mock, infected with WT HSV-1 (17syn+ strain) or inoculated with HSV-1 Δ34.5 (F strain) (*n* = 12/group). EAE was induced 30–35 days post-treatment and 4 mice from each group were euthanized at day 14, 21 or 25 post-EAE induction. **(A)** Representative images of lumbar sections of spinal cords stained with Luxol Fast Blue showing tissue demyelination. **(B)** Representative images of Luxol Fast Blue staining contrasted with Cresyl violet showing cellular infiltration. Myelin staining is observed in blue in the white matter and cell nuclei are colored purple. **(C)** Representative images of immunohistochemistry performed against the MBP protein. Image magnifications are 10× (left) and 20× (right) and correspond to day 21 post-EAE induction. **(D)** Quantitative histopathological analyses of spinal cord lumbar sections. Values represent the mean ± SEM of three independent experiments (4 mice/group per day evaluated). Data were analyzed using one-way ANOVA with Dunnett's multiple comparison post-test; **p < 0.01; n.s. non-significant. **(E)** Representative western blot images for MBP (upper panel) and β-actin (lower panel) in the spinal cord at day 14 post-EAE induction. The graph shows densitometric analyses for MBP bands that were normalized to β-actin. Data represent the mean ± SEM of two independent experiments (*n* = 6). Comparisons between ratios were performed using one-way ANOVA with Dunnett's multiple comparison post-test; *p < 0.05.

On the other hand, as shown in Figures 4A–C, histology analyses of spinal cord tissues revealed morphological alterations that were more evident for the experimental group infected with the Δ34.5 mutant virus and induced to undergo EAE, as evidenced after staining with Luxol Fast Blue and performing MBP immunohistochemistry. In these animals, this tissue displayed significant cellular infiltration and loss of myelin, consistent with more severe EAE than the other groups (Figure 4D). Importantly, histological samples of mice infected with WT HSV-1 and treated to undergo EAE did not display significant alterations, possibly because these animals experienced fewer maximum disease scores than the Δ34.5 mutant virus-inoculated group. Moreover, western blot analysis of MBP protein expression in the spinal cord showed reduced expression of this CNS protein in both groups inoculated with HSV-1, which was significantly lower in the Δ34.5 mutant virus-inoculated group than the mock-infected mice (Figure 4E).

Taken together, these results indicate that asymptomatic infection with HSV-1 either, with a WT virus or mutant virus that cannot replicate in neurons significantly affects the outcome of EAE, suggesting a direct relationship between both, the virus and this autoimmune disease.

### Asymptomatic HSV-1 Infection Increases EAE-Associated Inflammation

**Figure 5:**
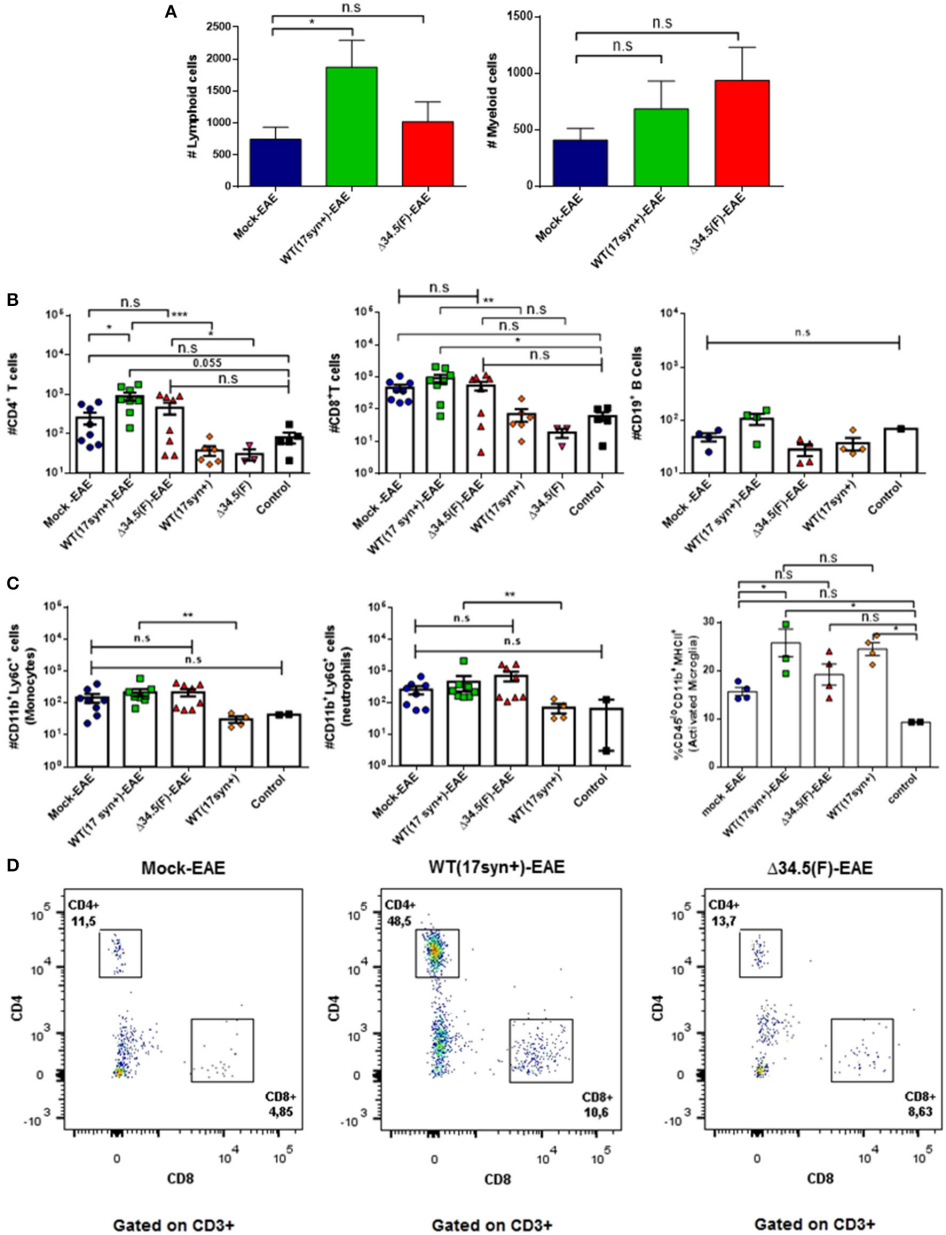
Animals infected with WT HSV-1 and treated to undergo EAE show increased numbers of CD4^+^ T cell infiltrating the brain. Mice were mock-treated, infected with WT HSV-1 (17syn+ strain) or inoculated with HSV-1 Δ34.5 (F strain). EAE was induced 4 weeks post-HSV-1 inoculation. At day 14 post-EAE induction, mice were perfused and the brain was harvested and processed to isolate immune infiltrating cells. Infected and uninfected mice without EAE were included as controls. **(A)** Total lymphoid cells (left) and myeloid cells (right) infiltrating the brains of mice induced to develop EAE. Values represent the means ± SEM of two independent experiments. Data were analyzed with one-way ANOVA with Dunnett's multiple comparison post-test; *p < 0.05 (*n* = 8/group). **(B)** Infiltrating CD4^+^ (left) and CD8^+^ (middle) T cells, or CD19^+^ (right) B cells in the brains of mice induced to develop EAE and uninfected or inoculated with HSV-1, yet without EAE induction are plotted individually. **(C)** Infiltrating Ly6C^+^ (left) and Ly6G^+^ (middle) myeloid cells in the brains of mice induced to develop EAE and either uninfected or inoculated with HSV-1, yet without EAE induction are plotted individually. Data are means ± SEM of two independent experiments for the EAE group (*n* = 8/group) or without EAE group (*n* = 2–5/group). For the percentage of activated CD45^lo+^/CD11b^+^/MHC-II^+^ microglia (right), the data are means ± SEM of *n* = 4/group. Data were analyzed using Kruskal–Wallis and Dunn's multiple comparisons post-test; ***p < 0.001, **p < 0.01, *p < 0.05, and n.s. non-significant. *T*-tests were used to compare animals inoculated with either, WT or Δ34.5 HSV-1, induced to develop EAE and their counterparts without EAE. **(D)** Representative FACS plots showing the distribution of lymphoid T cells in the brain. Live single cells were pre-gated over CD3^+^ and CD19^+^ cells. CD3^+^ T cells were subdivided into CD4^+^ and CD8^+^ T cells.

To determine if previous asymptomatic infection with HSV-1 favors the infiltration of immune cells into the CNS after EAE is induced, we performed flow cytometry analyses of the brain and spinal cord at day 14 post-EAE induction and assessed the presence of CD4^+^ T cells (CD3^+^/CD4^+^ cells), CD8^+^ T cells (CD3^+^/CD8^+^ cells), and B cells (CD19^+^ cells), as well as myeloid cells, namely monocytes (CD45hi^+^/CD11b^+^/Ly6G^+^ cells), neutrophils (CD45hi^+^CD11b^+^Ly6G^+^ cells), and activated microglia (CD45^lo+^/CD11b^+^/MHC-II^+^). As shown in Figure 5A, the brains of mice infected with WT HSV-1 and induced to undergo EAE displayed significantly more cellular infiltration of lymphoid cells than the uninfected group. In contrast, those animals previously infected with the Δ34.5 mutant virus had increased infiltration of myeloid cells in this tissue, although the differences were not statistically significant. Because HSV-1 latent brain infection has been reported to be accompanied by persistent T cell infiltration (13, 15), we sought to determine if this would be the case in our HSV-1-EAE model. As controls, mice infected with the WT or the mutant virus alone, without EAE induction, were evaluated at equivalent time-points as mice that were inoculated with the virus and then treated to undergo EAE (6 weeks post-infection) in order to evaluate the amounts of T cells in the brain or spinal cord as compared to healthy mice. Additionally, mice infected with the WT virus without EAE induction were evaluated to assess myeloid cell infiltration. Surprisingly, mice infected with WT virus and treated to undergo EAE displayed a significantly higher number of CD4^+^ T cells in the brain as compared to the mock-EAE group (Figures 5B,D). Moreover, when we compared the group infected with the WT virus with EAE induction with its counterpart without EAE induction, we observed a significant increase in the infiltration of CD4^+^ T cells after the initiation of EAE (Figure 5B). The antigen specificity of these T cells is unknown and needs to be further evaluated in future assays as they could be MOG-specific or HSV-1-specific.

On the other hand, although an increase in CD8^+^ T cells was observed after the induction of EAE in the WT HSV-1-infected animals, the differences were not significantly higher than those seen in the mock-infected animals undergoing EAE (Figure 5B). However, neutrophils and monocytes were significantly augmented in WT HSV-1 infected-EAE induced animals as compared to animals inoculated with the WT virus without EAE induction (Figure 5C). Nevertheless, there no were significant differences for these cell populations between WT HSV-1 infected-EAE induced animals and control uninfected mice (healthy) (Figure 5C).

Regarding activated microglia expressing the MHC-II surface marker, the percentage of these cells in the brain was higher in the WT HSV-1-EAE group than the mock-EAE group (Figure 5C). However, these differences seem to be an effect due latent brain infection by HSV-1 rather than EAE induction, as a similar result was observed in HSV-1 WT-infected mice without EAE, as compared to control mice. Overall, there were no significant increases in the number of these cells in the brain between the HSV-1 WT-EAE group and HSV-1 WT-infected mice without EAE (Figure 5C). This finding is consistent with a previous study that reported prolonged microglia cell activation following experimental herpes encephalitis (13).

**Figure 6:**
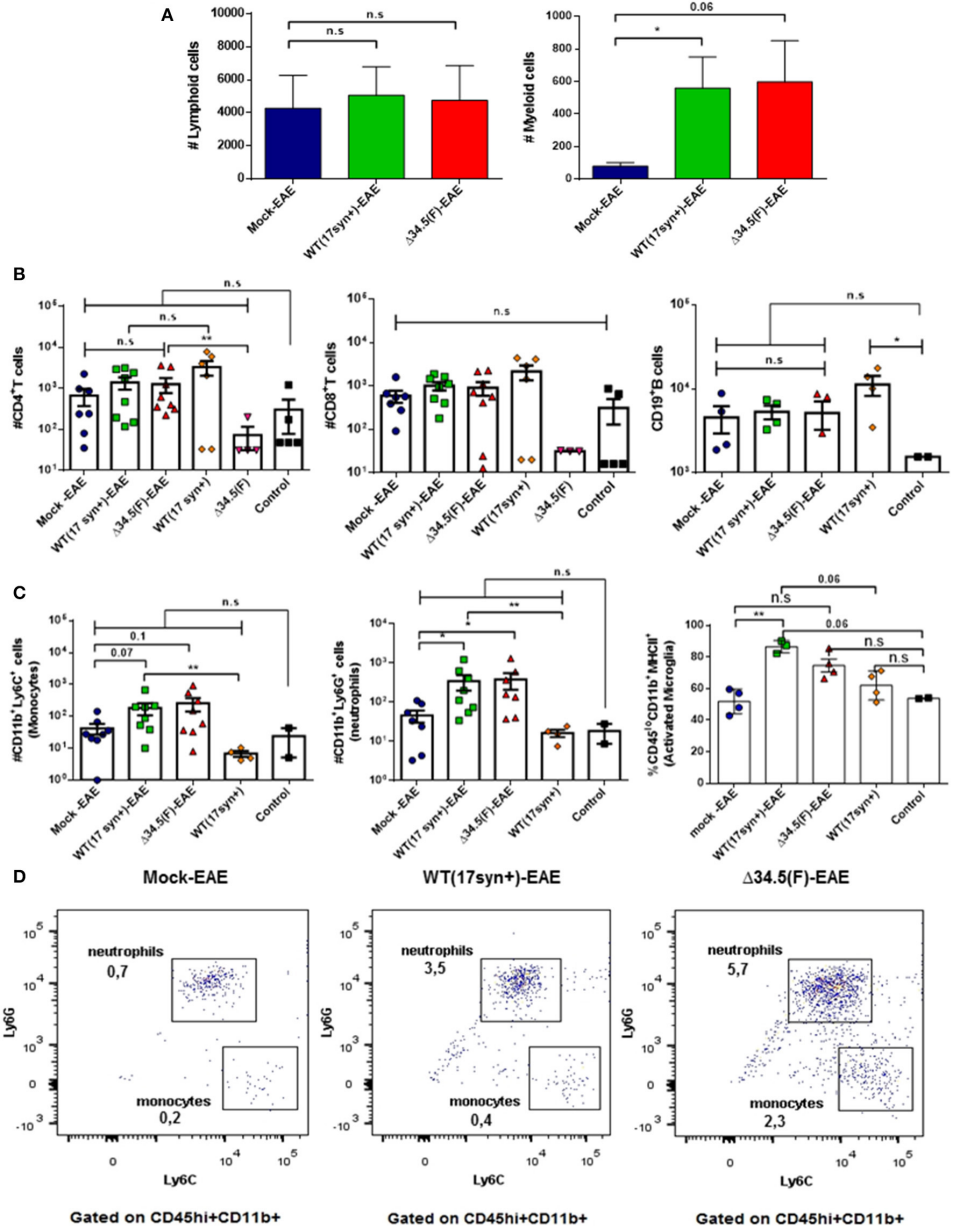
Animals inoculated with HSV-1 and treated to undergo EAE display increased numbers of neutrophils infiltrating the spinal cord. Mice were mock-treated, infected with WT HSV-1 (17syn+ strain) or inoculated with HSV-1 Δ34.5 (F strain). EAE was induced 4 weeks post-infection. At day 14 post-EAE induction, mice were perfused, and the spinal cords were harvested and processed to isolate immune cells infiltrating this tissue. Virus-inoculated and uninfected mice without EAE were included as controls. **(A)** Total lymphoid cells (left) and myeloid cells (right) infiltrating the spinal cords of mice induced to develop EAE. Values represent the means ± SEM of two independent experiments. Data were analyzed using Kruskal–Wallis and Dunn's multiple comparisons post-test; *p < 0.05 (*n* = 8/group). **(B)** Infiltrating CD4^+^ (left) and CD8^+^ (middle) T cells, or CD19^+^ B cells (right) from the spinal cord of mice induced to develop EAE and uninfected or inoculated with HSV-1, yet without EAE are plotted individually. **(C)** Infiltrating Ly6C^+^ (left) and Ly6G^+^ (middle) myeloid cells from the spinal cords of mice induced to develop EAE and uninfected or mice inoculated with HSV-1, yet without EAE are plotted individually. Data are means ± SEM of two independent experiments for the EAE group (*n* = 8/group) and without EAE group (*n* = 2–5/group). For the percentage of CD45^lo+^/CD11b^+^/MHC-II^+^ activated microglia (right), the data are means ± SEM of *n* = 4/group. Data were analyzed using Kruskal–Wallis and Dunn's multiple comparisons post-test **p < 0.01, *p < 0.05, and n.s. non-significant. *T*-tests were used to compare animals inoculated with either, WT or Δ34.5 HSV-1, induced to develop EAE and their counterparts without EAE. **(D)** Representative FACS plots showing the distribution of infiltrating myeloid cells in the spinal cords. Live single cells were pre-gated on CD45^+^ and CD11b^+^ cells. Infiltrating CD45^hi+^/CD11b^+^ myeloid cells were subdivided into neutrophils (Ly6G^+^) and monocytes (Ly6C^+^).

Regarding the spinal cord, overall no significant differences were observed in the number of infiltrating lymphoid cells between the infected groups and the mock-treated group induced to develop EAE (Figure 6A), and only a significant increase of CD4^+^ T cells was observed for the HSV-1 Δ34.5-EAE group as compared to HSV-1 Δ34.5-inoculated mice without EAE induction (Figure 6B). Nevertheless, HSV-1-inoculated mice treated to experience EAE had a greater number of infiltrating myeloid cells as compared to the mock-EAE group (Figures 6A,C), which were mainly neutrophils as shown in Figures 6C,D. Moreover, while the number of neutrophils and monocytes were similar between control mice and WT-infected mice without EAE, these cells were significantly augmented in the HSV-1 WT-EAE group as compared with animals without EAE (Figure 6C). Finally, the amount of activated microglia in the spinal cord of WT-infected mice induced to develop EAE was significantly higher than that in the mock-EAE group, as well as in WT-infected mice without EAE, and healthy mice (Figure 6C).

**Figure 7:**
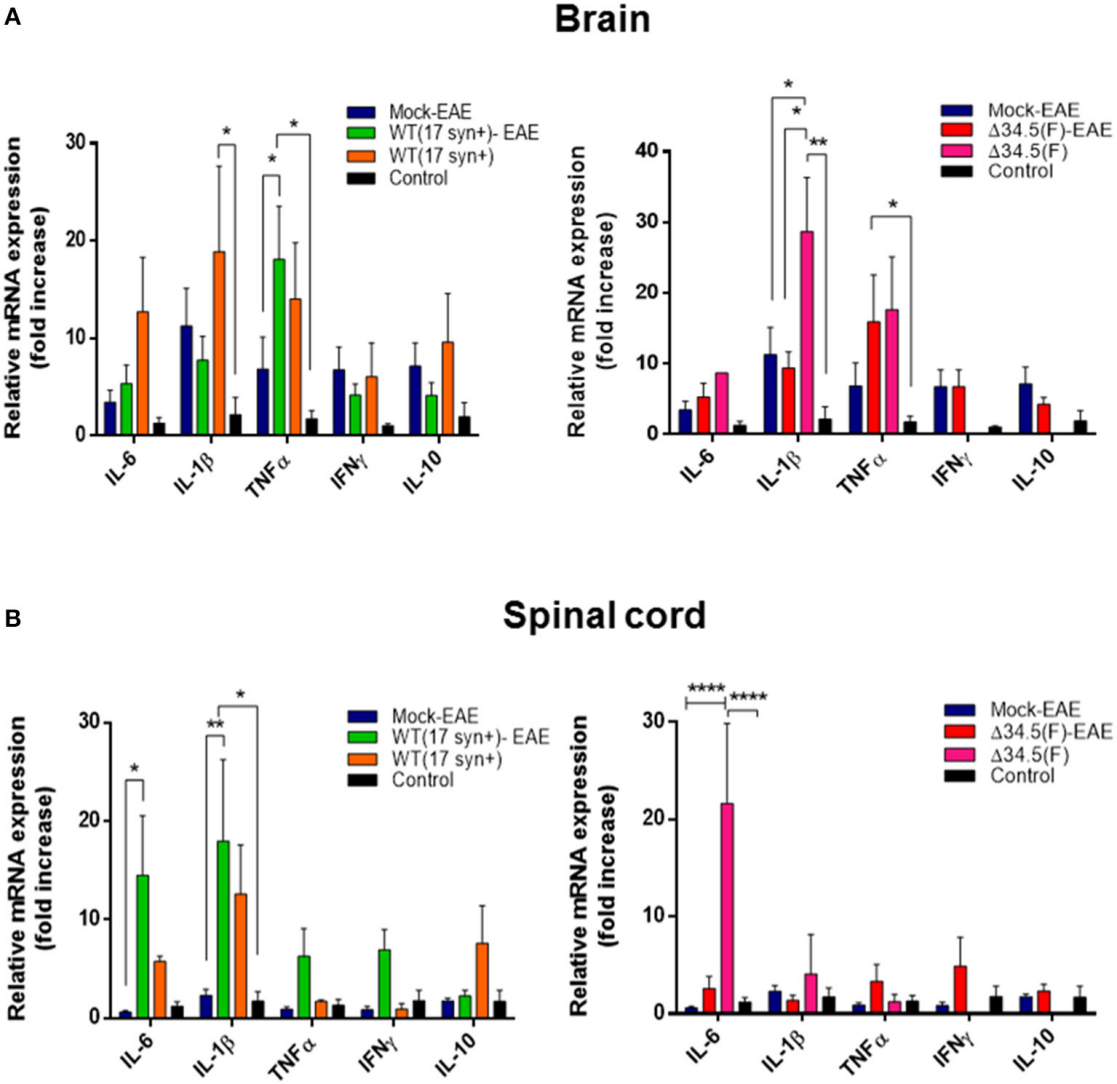
Asymptomatic HSV-1 infection increases the expression of pro-inflammatory cytokines in the brain and spinal cord. Mice were mock-treated, infected with WT HSV-1 (17syn+ strain) or inoculated with HSV-1 Δ34.5 (F strain). Four weeks post-virus inoculation EAE was induced. Fourteen days post-EAE induction, brain, and spinal cord homogenates were evaluated by RT-qPCR to assess cytokine expression at the mRNA level using the 2^−ΔΔCT^ method with β-actin as a reference gene. Virus-inoculated and unifected animals without EAE were also included. **(A)** Relative expression levels of proinflammatory cytokines IL-6, IL1-β, TNF-α, and IFN-γ, and the anti-inflammatory cytokine IL-10 in the brain of WT HSV-1 (17syn+ strain)-infected mice (left) and Δ34.5(F)-infected mice (right), compared to the mock-EAE group. **(B)** Relative expression levels of cytokines in the spinal cord of WT (17syn+ strain)-infected mice (left) and HSV-1 Δ34.5 (F strain)-inoculated mice (right), compared to the mock-EAE group. Values represent means ± SEM of two independent experiments for the EAE group (*n* = 8/group) and group without EAE (*n* = 3–4/group). Data were analyzed using two-way ANOVA followed by Tukey's post-test; ****p < 0.0001, **p < 0.01, and *p < 0.05.

Next, to evaluate whether asymptomatic infection with HSV-1 modulates the cytokine environment in the CNS upon EAE induction, we performed RT-qPCR for a set of cytokines that either, promote an inflammatory state in this tissue (i.e., IL-1β, IL-6, TNF-α and IFN-γ) or an anti-inflammatory environment (i.e., IL-10). Mice infected with WT or the mutant virus alone without EAE induction and healthy uninfected mice were included as controls. As shown in Figure 7A, the brain of mice infected with WT HSV-1 displayed a proinflammatory environment, even in the absence of EAE induction, which was mainly characterized by a significant increase in IL-1β mRNA. Moreover, increased levels of TNF-α mRNA were expressed in the brain of WT-infected animals with EAE induction compared to equivalent tissue obtained from mice induced to develop EAE without a previous HSV-1 infection (Figure 7A). Likewise, IL-1β and TNF-α mRNA levels were also significantly increased in the brains of mice infected with the mutant virus, without EAE induction or induced to undergo EAE, respectively (Figure 7A). Notably, IL-1β mRNA expression was significantly higher in the Δ34.5 HSV-1-inoculated animals as compared to uninfected mice induced to develop EAE (Figure 7A). Importantly, a chronic proinflammatory environment in the brain induced by previous HSV-1 exposure, before EAE initiation could predispose that animals to develop a more severe EAE disease. Cytokine mRNAs levels in the spinal cord displaying important variations, as compared to mock-infected animals, were IL-6 and IL-1β in the WT HSV-1-EAE group as compared to mock-inoculated mice with EAE (Figure 7B), and IL-6 in the Δ34.5 HSV-1-infected group without EAE as compared to other groups with EAE, as well as healthy mice, as shown in Figure 7B.

### Asymptomatic Mice Infected With WT HSV-1 Display Increased Amounts of anti-HSV-1 Antibodies After EAE Induction

**Figure 8:**
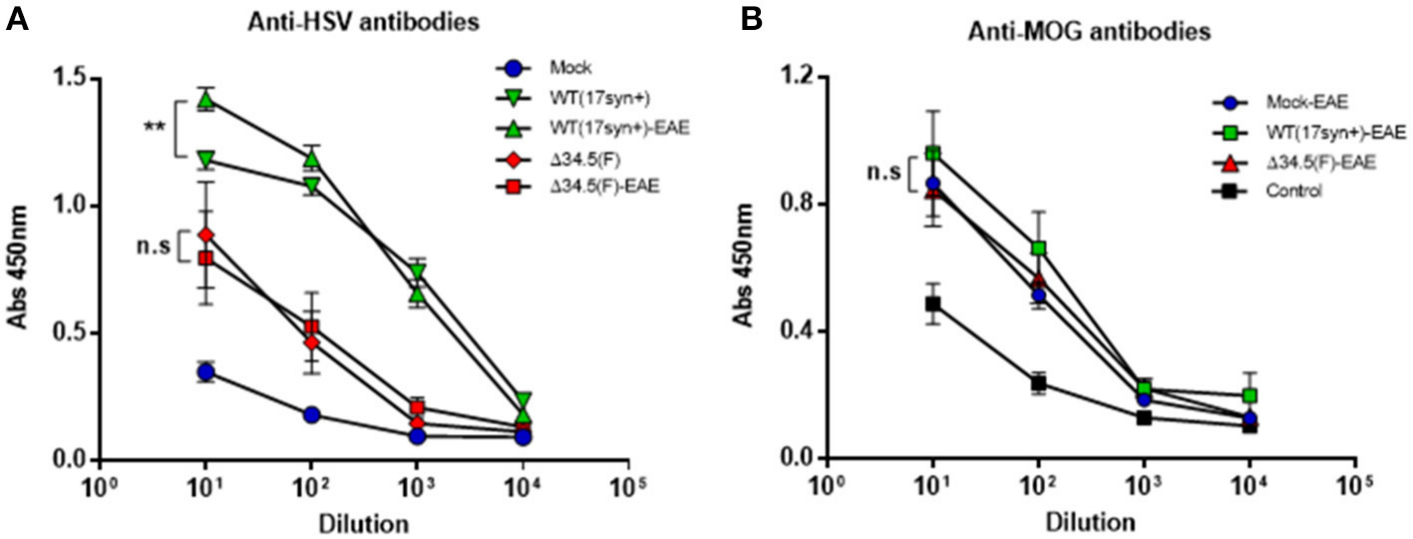
Animals infected with WT HSV-1 and then treated to undergo EAE display increased anti-HSV antibodies after EAE induction. Mice were mock-treated (blue), infected with WT HSV-1 (17syn+ strain, green), or inoculated with HSV-1 Δ34.5 (F strain, red). EAE was induced in the indicated groups (EAE) 4 weeks post-virus inoculation. At day 14 post-EAE induction, sera were harvested and the levels of **(A)** anti-HSV-1 IgG antibodies (*n* = 8/group) and **(B)** anti-MOG IgG antibodies (*n* = 10/group) were quantified using ELISA. Data are means ± SEM of two independent experiments. Data were analyzed using two-way ANOVA followed by Tukey's post-test; **p < 0.01 and n.s. non-significant.

Given the results obtained above, it is possible that asymptomatic infection with HSV-1 predisposes the animals to undergo increased EAE severity, but it is also possible that the induction of EAE in previously-infected animals may promote virus reactivation in the CNS or periphery and facilitate enhanced neurodegenerative disease. To preliminarily assess this latter scenario, we quantified the amount of viral DNA in the brain and trigeminal ganglia of each group 45 days after HSV-1 inoculation, which is corresponds to 15 days post-EAE induction for the EAE group. Interestingly, no significant variations in the loads of viral DNA were observed at this time-point between animals that were synchronously inoculated with HSV-1, and then treated or not to undergo EAE (Supplementary Figure 2). Additionally, we assessed the amounts of circulating antibodies against HSV-1 in the sera of virus-inoculated animals before- and 14 days after-EAE induction. Interestingly, we found that those animals that were previously infected with WT HSV-1 and then treated to undergo EAE displayed a modest, yet significant increase in the quantity of anti-HSV-1 antibodies in the serum (Figure 8A). Although these differences are not substantial, this result suggests possible viral reactivation at the molecular level (expression of HSV-1 antigens without the release of new infectious particles), which requires further attention. Alternatively, EAE induction may facilitate immune cell infiltration into the CNS and increased access to HSV-1 antigens.

Additionally, we assessed the quantity of MOG-specific antibodies in the sera of animals inoculated or not with HSV-1 and then treated to undergo EAE. As shown in Figure 8B, although mice inoculated with WT HSV-1 displayed significantly higher amounts of anti-MOG antibodies after EAE induction as compared to control healthy mice, no significant differences were observed between the animals of the WT HSV-1-EAE, Δ34.5 HSV-1-EAE, or mock-EAE groups (Figure 8B).

## Discussion

Infections with human herpesviruses have been suggested as potential triggers or enhancers of MS in recent reports (21, 23), yet studies that assess or support a role for HSV-1 infection are relatively scarce and a direct relationship between this virus and MS disease has not been reported before (25, 46–48). Although the fact that HSV-1 infects the CNS makes this virus a suspect candidate in MS, the fact that HSV-1 infection is highly prevalent in the human population, unlike MS which is significantly less frequent, somewhat argues against this idea. However, asymptomatic HSV-1 infection in the CNS may be insufficient for developing MS *per se* and the initiation of the disease likely requires other contributing elements, such as genetic and environmental factors (49–51). Importantly, the prevalence of CNS infection with HSV-1 in otherwise healthy individuals is somewhat unknown, as this is not a routine analysis performed after death. Despite the fact that CNS infection with HSV-1 in healthy individuals is underdetermined, it is possible to foresee that the chances of developing HSV-1 infection of the CNS will likely increase with aging, as progressive senescence of the immune system may allow HSV-1 to reactivate from peripheral tissues, such as the trigeminal ganglia and access the brain spreading within this tissue (10, 11, 52). Furthermore, repeated HSV-1 reactivations throughout the life of an individual may provide opportunities for increased number of neurons to be infected with this virus as a person ages. Additionally, neuronal senescence may also facilitate neurodegenerative disorders by HSV-1 and eventually facilitate MS initiation and progression (12, 15).

Here, we observed that asymptomatic infection with HSV-1 after intranasal virus inoculation can predispose the host to an earlier onset and more severe EAE disease. Our results showed a significant increase in the demyelination of spinal cords and brain in animals previously inoculated with HSV-1, which was more evident for those treated with Δ34.5 mutant virus. Surprisingly, these results suggest that viral replication in the brain may not be necessary for experiencing increased EAE severity after exposure to HSV-1.

Although we did not observe significant histological alterations in brain samples obtained from mice that displayed an earlier onset of EAE, or increased EAE severity upon a previous inoculation with WT-HSV-1, several molecular markers associated with inflammation and cellular infiltration in the CNS of these animals could be detected by other means. As reported above, we found that IL-6 mRNA was elevated in the spinal cord of infected animals with EAE as compared to mock-EAE treated mice. Importantly, this cytokine has been reported to be a key player in the development of autoimmune diseases by inhibiting the induction of regulatory T cells (Tregs) (53, 54). Studies performed in humans with relapsing-remitting MS show that IL-6 supports T cell effector function resistance to regulation by Tregs, which may contribute to disease severity (55). Moreover, the elevated levels of TNF-α and IL-1β mRNA observed in the brain and spinal cord of infected mice may also promote BBB permeability, possibly through mechanisms previously reported over astrocytes by IL-1β or through effects over adhesion molecules and chemokines by TNF-α leading to BBB damage (56–58). An interesting finding was the fact that the BBB of asymptomatic HSV-1-inoculated mice remained permeable to the Evans Blue dye 30 days after virus exposure. Although alterations in the BBB during HSV-1 infection had been reported before, this phenomenon was only observed in *in vitro* BBB models, or during acute CNS infection with this virus (HSV-1 encephalitis), but not during asymptomatic infection (42–44). Our results show that the disruption of the BBB occurs independent of encephalitis and persists in the absence of infectious virus in the CNS. Moreover, these results suggest that intranasal virus inoculation is enough to disrupt the BBB for a long period. However, it remains to be determined how long these alterations last and whether they are key for the observations reported herein.

On the other hand, while CD4^+^ T cells have been shown to play a key role in EAE onset and severity (59), and that we observed that these cells were increased in the brain of WT HSV-1-EAE treated mice, relevant roles for other immune cells, such as neutrophils are emerging as an immune component contributing to CNS damage (60–62). Importantly, we found that these cells were increased in the CNS of the experimental groups inoculated with HSV-1, as compared to mock-treated mice. This finding contributes to the notion that neutrophils play a detrimental role in EAE and eventually MS pathogenesis. Additionally, it will be interesting to assess the contribution and role of virus-specific CD4^+^ and CD8^+^ T cells in these experiments, as these cells may be contributing to CNS inflammation by promoting immune cell access to the CNS, enhance cytokine secretion in these tissues or mediate direct neuron damage (63). Importantly, previous reports suggest that virus infection can increase the susceptibility to autoimmune diseases by eliciting bystander inflammation and the activation of autoreactive cells, which can lower the threshold for disease development (64, 65).

Although our findings suggest a role for asymptomatic brain infection by HSV-1 on the onset and severity of MS, it remains unknown whether EAE induction in these animals reactivates HSV-1 at the molecular level, a process that is characterized by viral antigen expression without detectable infectious virus (18, 66). The fact that animals inoculated with HSV-1 and then treated to undergo EAE displayed increased amounts of anti-HSV-1 antibodies in the sera suggests that HSV-1 molecular reactivation may be occurring in these mice, although this remains to be further assessed, as EAE may also be promoting the infiltration of immune cells into this tissue increasing their access to HSV-1 antigens. Importantly, because the mutant virus elicited enhanced EAE symptoms, even more than the WT virus for some of the analyzed parameters, it is also possible that the main mechanism behind enhanced EAE by previous HSV-1 inoculation may be a consequence of a long-lasting imprinting of the virus over infected cells early after virus inoculation, or even adjacent cells, which could trigger an inflammatory response that increases the host susceptibility to undergo this autoimmune disease with increased severity (63).

Interestingly, Whitley et al. (29) have previously reported that the Δ34.5-mutant virus assessed herein has lost most of its capacity to spread from the nasal mucosae to the CNS and replicate in this latter tissue, and displayed a reduced ability to establish latency and reactivate *ex vivo*, which is consistent with the observations made herein, with only a fraction of animals displaying virus in the brain or trigeminal ganglia at very low levels. This fact suggests that the enhanced severity of EAE observed in our experiments, after asymptomatic HSV-1 infection may be due to an inflammatory signature imprinted in the infected tissues early after infection, rather than an effect of latent virus in the nervous system or potential viral reactivation from this tissue as this mutant virus is limited in this aspect. This notion is supported by previous studies that reported increased susceptibility to severe EAE in mice after viral infections were cleared (63, 67). One of these studies reported that a transient brain viral infection induces the formation of tissue-resident memory T cell (TRM) clusters that elicit a persisting CCL5 chemotactic signal, which contributed to increased autoimmune lesions in the brain by a virus-independent mechanism after EAE induction (63). In the other case, long-term exacerbated EAE was observed in mice after a previously resolved influenza infection (67). On the other hand, our results somewhat differ from those reported for another herpesvirus, namely herpesvirus γHV-68, a murine homolog of EBV. Animals infected with this virus showed an earlier onset, and a worse clinical EAE outcome that was accompanied with enhanced T cell infiltration within the CNS and a potent Th1 response (22). This was reversed when the animals were infected with a virus deficient in the establishment of latency (23).

Given the existence of antivirals that are specific for herpesviruses, such as acyclovir, at first it is tempting to speculate that such compounds may delay the onset of EAE in animals inoculated with HSV-1, or reduce the severity of the disease in these mice once initiated. However, because the Δ34.5 mutant virus is attenuated in neurons with a reduced capacity to establish latency, and that the animals inoculated with this virus displayed more severe EAE than with the WT virus, the use of such drugs may not necessarily have therapeutic effects. Consistent with this notion, we did not observe an increase in viral DNA loads in the brain or trigeminal ganglia after EAE induction, suggesting that HSV-1 would not be replicating during this stage of the disease in the virus-infected animals. Nevertheless, it will be interesting to perform the experiments carried out in this study in the presence of drugs such as acyclovir after virus infection to determine the potential contribution of HSV-1 replication before EAE onset, or at other stages of EAE that were not assessed herein.

Taken together, we report that a previous asymptomatic HSV-1 infection enhances EAE disease severity and onset, even in the absence of latent or reactivating virus. The mechanism mediating this relationship remains to be fully determined, but may be partially mediated by BBB disruption and the promotion of a virus-induced inflammatory environment in the CNS that is permissive for autoimmunity. These aspects call for additional investigations oriented at elucidating the role of HSV-1 over MS in humans, which eventually may lead to the identification of new pharmacological targets to treat or prevent the progression of this autoimmune disease.

## Data Availability Statement

The original contributions presented in the study are included in the article/Supplementary Material, further inquiries can be directed to the corresponding author/s.

## Ethics Statement

The animal study was reviewed and approved by Scientific Ethical Committee for Animal and Environmental Care of the Pontificia Universidad Católica de Chile.

## Author Contributions

LD, MA-L, JT-G, MO, MD, RN, CM, and OV contributed with experiments. LD, CR, SB, AK, and PG contributed to the design of the study and the formal analysis. All authors approved the submitted version. All authors contributed to the writing and editing of the manuscript.

## Conflict of Interest

The authors declare that the research was conducted in the absence of any commercial or financial relationships that could be construed as a potential conflict of interest.

## Supplementary Material

Supplementary Figure 1 Asymptomatic brain infection after intranasal inoculation with HSV-1. C57BL/6 mice were intranasally mock-treated, infected with WT HSV-1 (17syn+ strain), or inoculated with Δ34.5 HSV-1 (F strain) and weighted daily for 30 days. **(A)** Weight curves of infected and non-infected mice. Values represent means ± SEM from three independent experiments (*n* = 12/group). Data were analyzed using two-way ANOVA followed by Dunnett's multiple comparisons post-test; ^**^*p* < 0.01, ^*^*p* < 0.05. **(B)** HSV-1 *UL30* gene copies per gram of brain or trigeminal ganglia in a subset (*n* = 4/3 animals) of WT HSV-1 (17syn+ strain)-infected and HSV-1 Δ34.5 (F strain)—infected mice up to 30 days post-infection (values normalized to uninfected mice). Data were analyzed using two-way ANOVA followed by Tukey's post-test; ^**^*p* < 0.01.

Supplementary Figure 2 Viral DNA loads do not increase in the brain or trigeminal ganglia 15 days post-EAE induction. C57BL/6 mice were intranasally infected with WT HSV-1 (17syn+ strain), intranasally inoculated with Δ34.5 HSV-1 (F strain), or mock-treated and then induced to undergo EAE (in the indicated groups: EAE) 30 days post-virus inoculation. The animals were sacrificed 15 days post-EAE induction (45 days after virus inoculation) and DNA was extracted from the brain and trigeminal ganglia. The HSV-1 gene encoding UL30 was amplified by qPCR to quantify the number of copies of the viral genome in the tissue. The values were normalized based on mock-treated mice. Data were analyzed using two-way ANOVA followed by Tukey's post-test (n.s. non-significant).
