# Supplementary figures and images for: Asymptomatic Herpes Simplex Virus Type 1 Infection Causes an Earlier Onset and More Severe Experimental Autoimmune Encephalomyelitis
Source: Front Immunol. 2021 Feb 15;12:635257. doi: 10.3389/fimmu.2021.635257 (PMC7928309; doi:10.3389/fimmu.2021.635257)

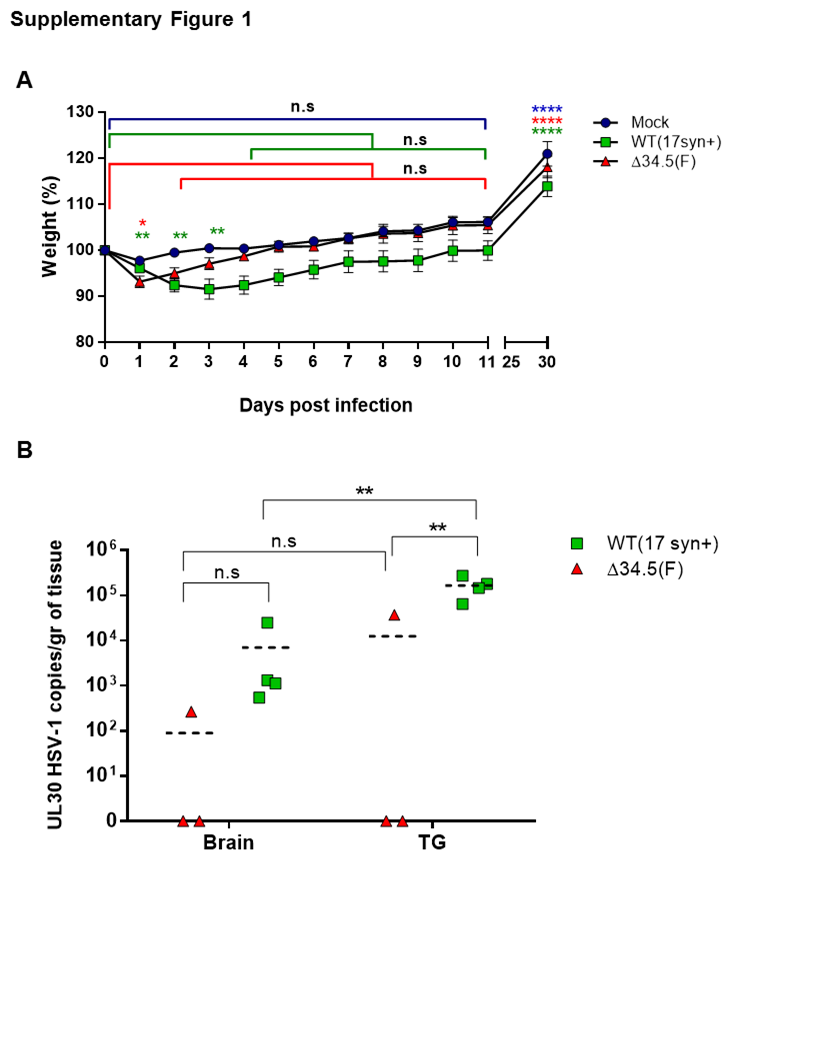

Supplement: Supplementary Figure 1 — Asymptomatic brain infection after intranasal inoculation with HSV-1. C57BL/6 mice were intranasally mock-treated, infected with WT HSV-1 (17syn+ strain), or inoculated with Δ34.5 HSV-1 (F strain) and weighted daily for 30 days. (A) Weight curves of infected and non-infected mice. Values represent means ± SEM from three independent experiments (n = 12/group). Data were analyzed using two-way ANOVA followed by Dunnett's multiple comparisons post-test; **p < 0.01, *p < 0.05. (B) HSV-1 UL30 gene copies per gram of brain or trigeminal ganglia in a subset (n = 4/3 animals) of WT HSV-1 (17syn+ strain)-infected and HSV-1 Δ34.5 (F strain)—infected mice up to 30 days post-infection (values normalized to uninfected mice). Data were analyzed using two-way ANOVA followed by Tukey's post-test; **p < 0.01. [file Image_1.TIF]

## Slide 1
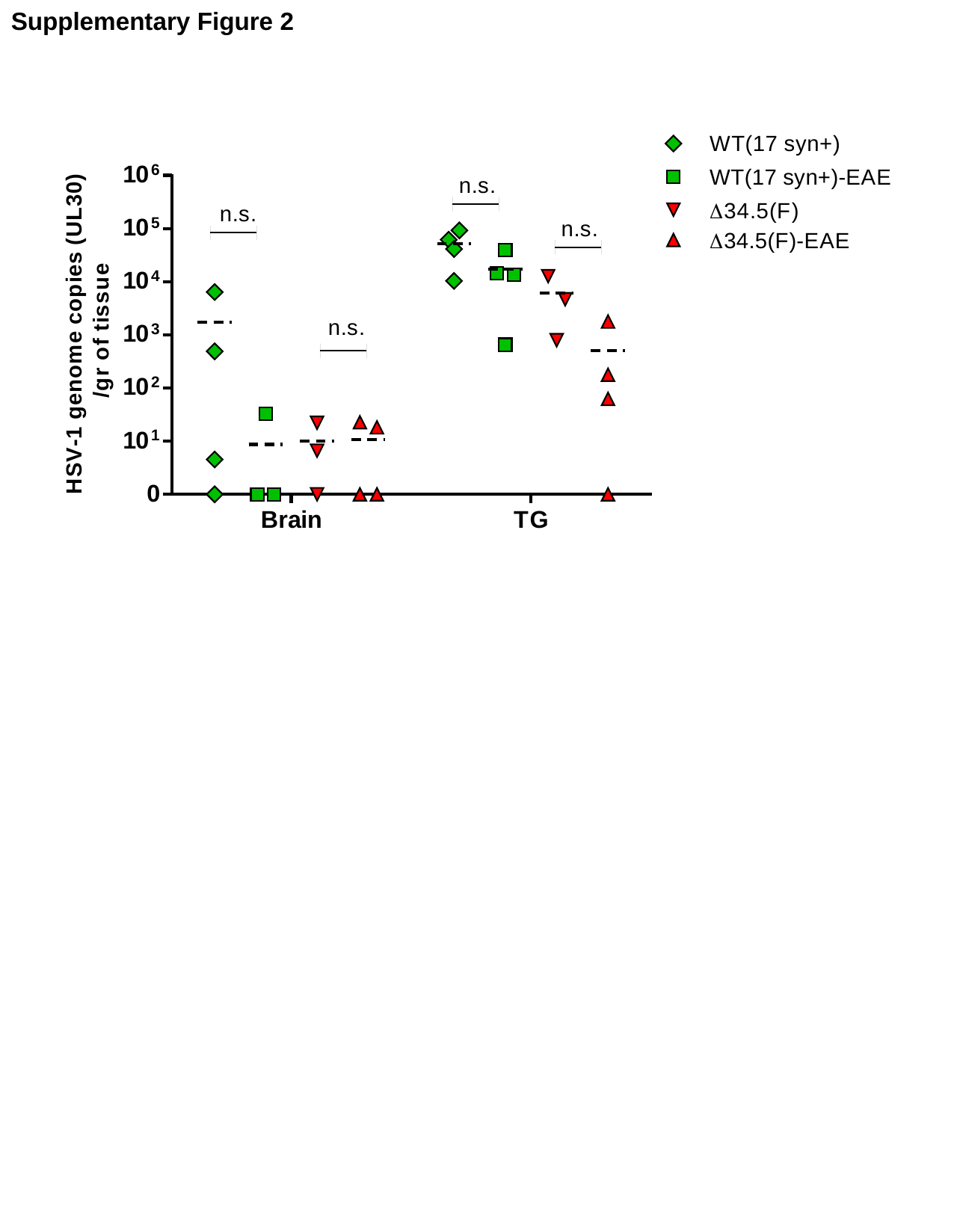

Supplementary Figure 2

Supplement: Supplementary Figure 2 — Viral DNA loads do not increase in the brain or trigeminal ganglia 15 days post-EAE induction. C57BL/6 mice were intranasally infected with WT HSV-1 (17syn+ strain), intranasally inoculated with Δ34.5 HSV-1 (F strain), or mock-treated and then induced to undergo EAE (in the indicated groups: EAE) 30 days post-virus inoculation. The animals were sacrificed 15 days post-EAE induction (45 days after virus inoculation) and DNA was extracted from the brain and trigeminal ganglia. The HSV-1 gene encoding UL30 was amplified by qPCR to quantify the number of copies of the viral genome in the tissue. The values were normalized based on mock-treated mice. Data were analyzed using two-way ANOVA followed by Tukey's post-test (n.s. non-significant). [file Presentation_1.PPTX]
